# A Human Rights-Based Approach To Acute Mental Health Crisis Care

**DOI:** 10.1192/j.eurpsy.2022.60

**Published:** 2022-09-01

**Authors:** L. Mahler

**Affiliations:** Clinics of Theodor-Wenzel-Werk, Department Of Psychiatrie And Psychotherapy, Berlin, Germany

## Abstract

The United Nations Convention on the Rights of Persons with Disabilities as well as the new guidance on community mental health services recently published by the World Health Organization formulate clear goals for the future of psychiatry and psychosocial support. Innovative concepts of psychiatric care that focus on full participation, recovery-orientation and the prevention of coercion play an important role in achieving these goals. Implementing and scientifically evaluating the effects of such models in mental health services needs to be prioritized in national mental health planning and budgeting decisions. In this lecture, Dr. Lieselotte Mahler will address the requirements of psychiatric concepts, specifically on acute psychiatric wards, to enable participation and prevent coercion. Using practical examples and evaluated models (e.g. Weddinger Modell, standardized post-coercion review sessions), she will make suggestions on how a psychiatry oriented towards human rights can also be implemented within existing clinical structures. Dr. Lieselotte Mahler will discuss, based on the current state of research as well as practical experience, which challenges exist with regard to patient autonomy and prevention of coercion in clinical practice and how these can be addressed from a human rights perspective.

**Disclosure:**

No significant relationships.

